# A Novel Rat Model to Study the Role of Intracranial Pressure Modulation on Optic Neuropathies

**DOI:** 10.1371/journal.pone.0082151

**Published:** 2013-12-18

**Authors:** Uttio Roy Chowdhury, Bradley H. Holman, Michael P. Fautsch

**Affiliations:** Department of Ophthalmology, Mayo Clinic, Rochester, Minnesota, United States of America; University of Illinois at Chicago, United States of America

## Abstract

Reduced intracranial pressure is considered a risk factor for glaucomatous optic neuropathies. All current data supporting intracranial pressure as a glaucoma risk factor comes from retrospective and prospective studies. Unfortunately, there are no relevant animal models for investigating this link experimentally. Here we report a novel rat model that can be used to study the role of intracranial pressure modulation on optic neuropathies. Stainless steel cannulae were inserted into the cisterna magna or the lateral ventricle of Sprague-Dawley and Brown Norway rats. The cannula was attached to a pressure transducer connected to a computer that recorded intracranial pressure in real-time. Intracranial pressure was modulated manually by adjusting the height of a column filled with artificial cerebrospinal fluid in relation to the animal’s head. After data collection the morphological appearance of the brain tissue was analyzed. Based on ease of surgery and ability to retain the cannula, Brown Norway rats with the cannula implanted in the lateral ventricle were selected for further studies. Baseline intracranial pressure for rats was 5.5±1.5 cm water (n=5). Lowering of the artificial cerebrospinal fluid column by 2 cm and 4 cm below head level reduced ICP to 3.7±1.0 cm water (n=5) and 1.5±0.6 cm water (n=4), a reduction of 33.0% and 72.7% below baseline. Raising the cerebrospinal fluid column by 4 cm increased ICP to 7.5±1.4 cm water (n=2) corresponding to a 38.3% increase in intracranial pressure. Histological studies confirmed correct cannula placement and indicated minimal invasive damage to brain tissues. Our data suggests that the intraventricular cannula model is a unique and viable model that can be used to study the effect of altered intracranial pressure on glaucomatous optic neuropathies.

## Introduction

Glaucoma, the leading cause of irreversible blindness, is a progressive neurodegenerative disease with a complex and incompletely understood pathophysiology[1–3]. All known subtypes of glaucoma share a characteristic degeneration and concomitant cupping of the optic nerve head, the extent of which is directly associated with visual field loss[1,4]. An increased intraocular pressure (IOP) is the most prevalent risk factor for glaucoma and is generally believed to be a primary causal factor for neuronal cell damage in the optic nerve head[1,5–7]. However, exceptions to this correlation between elevated IOP and glaucomatous optic nerve damage have been noted. For example, elevated IOP is not observed in normal tension glaucoma (NTG) although damage to the optic nerve still occurs[6]. Additionally, individuals with elevated IOP do not always develop glaucoma and optic nerve damage[8]. Therefore, it is difficult to explain optic nerve damage based on elevated IOP alone[5,9,10].

The optic nerve is anatomically situated within the subarachnoid space. It is affected by two pressurized compartments – (i) the intraocular space where IOP is generated by a balance between the production and removal of aqueous humor[1] and (ii) the subarachnoid space which is filled by cerebrospinal fluid (CSF) creating a pressure that is equivalent to intracranial pressure (ICP)[4,5,11,12]. These two anatomical regions are separated by the lamina cribrosa[12], a perforated area in the posterior sclera through which the nerve fibers of the optic nerve pass.

During glaucoma, damage to the optic nerve has been explained by two interlinked theories. The mechanical theory states that optic nerve damage is directly caused by the mechanical forces arising out of elevated IOP. Alternatively, the blood flow theory states that changes in ocular blood flow in and around the optic nerve from elevated IOP are involved in glaucomatous pathology[13,14]. Recent studies have added significant evidence to complement the mechanical theory by incorporating ICP as an important component in maintaining optic nerve health. According to these studies, ICP is lower in primary open angle glaucoma (POAG) and NTG patients when compared to normal controls, and a low ICP is now considered to be a risk factor for glaucoma[4,5,9,15–22]. These studies have also established that homeostatic conditions necessary for maintaining a normal optic nerve are produced by a pressure differential between IOP and ICP as well as thickness of the lamina cribrosa[4,5,9,15–18,21,22]. A change in the translaminar pressure differential by an elevated IOP and a lowered ICP (as in POAG) or a lowered ICP alone (as in NTG) will alter the pressure gradient and subject the optic nerve to a posteriorly directed force that may cause severe optic nerve cupping as seen during glaucoma[5,16,17]. The opposite of this is found in patients suffering from pseudotumor cerebri and ocular hypotony. In the case of pseudotumor cerebri, the ICP is greater than IOP and causes swelling (papilledema) of the optic nerve head. During ocular hypotony, the IOP is lower than normal and causes a similar anteriorly directed force on the lamina cribrosa and subsequent papilledema[5,23,24]. The role of the pressure differential across the lamina cribrosa may also explain the enigmatic “reversal of cupping” without gaining visual acuity. This has been observed in glaucoma patients with IOP maintained at lower than normal levels for 5 years[25].

While these reports suggest a homeostatic balance of the translaminar pressure gradient may be responsible for maintaining optic nerve health, the evidence supporting this is based on retrospective and prospective studies and not on direct experimental evidence due to lack of a suitable animal model. As a result, it is difficult to determine the clinical significance of these findings. In the current study we describe a novel method to reduce or increase ICP in live non-anesthetized rats for an extended time in a quantitative and reproducible manner. 

## Materials and Methods

### Animals

This study was carried out in strict accordance with the recommendations in the Guide for the Care and Use of Laboratory Animals of the National Institutes of Health. Prior to initiation of studies, animal protocol A20212 was approved by the Mayo Clinic Institutional Animal Care and Use Committee. All Brown Norway (retired breeders, age >8 months, weight >300 g) and Sprague-Dawley rats (retired breeders, age >8 months, weight >250 g) used in this study were purchased from Charles River Laboratories (Wilmington, MA). The rats were maintained under a 12 hour light and 12 hour dark cycle and received standard rodent chow and water *ad libitum*. Upon arrival, the rats were housed in a stress free environment for at least 5 days for acclimation.

### Intracranial cannula implantation

**Figure 1:**
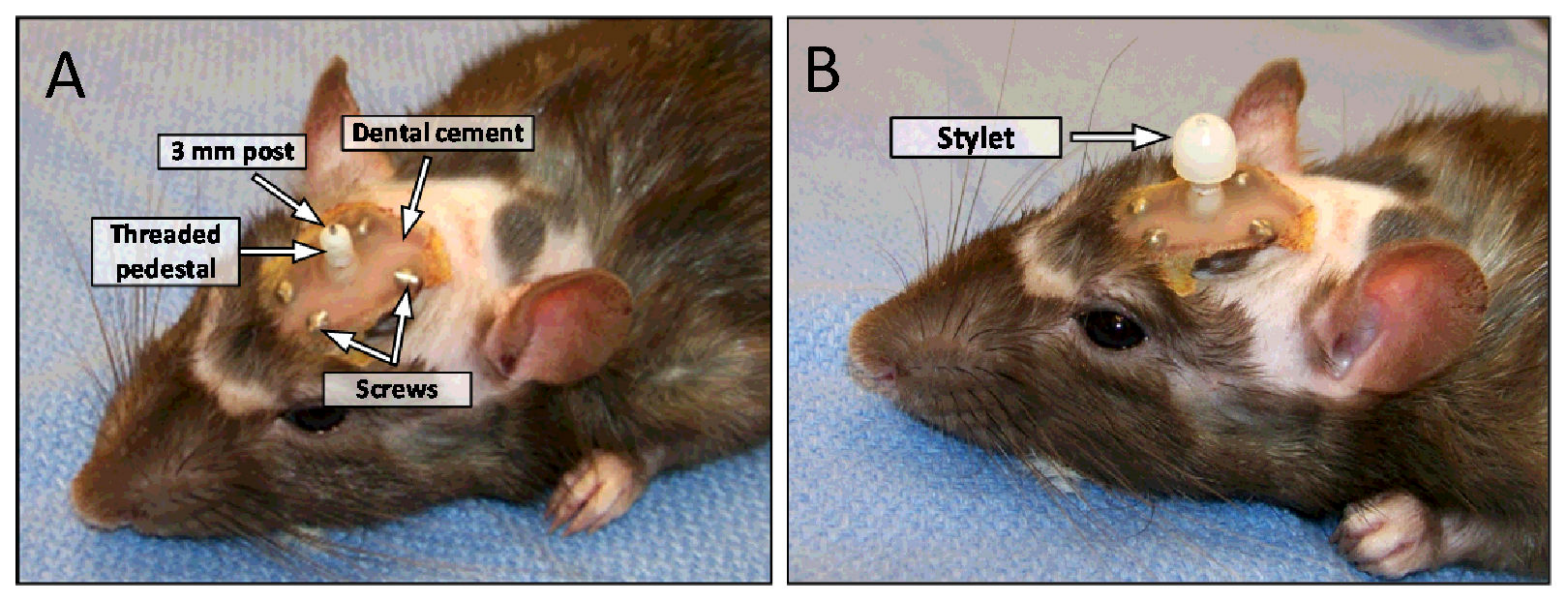
Representative picture showing implanted cannula after surgery. A) The stainless steel cannula was inserted through the guide cannula (enclosed by the threaded pedestal) and attached to the latter through a locking mechanism. The cannula assembly was secured to the skull with dental cement and 4 stainless steel screws fitted into the skull. The guide cannula had an external 3 mm post to which PE60 tubing was connected. B) Following surgery, a stylet was inserted and screwed onto the threaded pedestal of the guide cannula to prevent introduction of extraneous materials into the brain ventricle.

Cannula implantation was performed by Charles River Laboratories according to their established protocols and customized to meet our specific requirements. Briefly, rats were anesthetized using isoflurane, and a stainless steel internal cannula (also known as an injector or infusion cannula) of 20 gauge or 22 gauge bore size with an extended external post of 1.5 mm or 3 mm was inserted through the skull cap into the left lateral ventricle or the cisterna magna with the help of a rat stereotactic apparatus and guide cannula. A locking mechanism connected the internal cannula to the guide cannula. The guide cannula was provided with a threaded pedestal and extensions of various sizes to which PE tubing could be attached (Figure 1, A). Following cannula implantation, a stylet was inserted through the guide cannula extension and into the internal cannula to prevent introduction of extraneous materials and tissue debris (Figure 1, B). The stylet had a rounded plastic cap that screwed onto the threaded pedestal of the guide cannula (Figure 1, B). The entire cannula assembly was held in place by dental cement and four metal screws secured in the skull. Following surgery, the animals were allowed to recuperate for at least 5 days during which they received buprenorphine and cavprofen for pain management. Upon arrival at Mayo, animals were continuously supplied with a non-steroid anti-inflammatory drug (acetaminophen, Major Pharmaceuticals, Livonia, MI) in the drinking water (1mg/ml) for pain control secondary to subsequent ICP modulation. 

### Equipment and supplies for the rat intraventricular cannula (IVC) model

Implanted cannulae in the rats were attached to polyethylene (PE) tubing (Becton Dickinson, Franklin Lakes, NJ) and tethered to a swivel mount (Instech Solomon, Plymouth Meeting, PA) to allow head movement. The PE tubing was ultimately connected to a DTXplus pressure transducer (Argon Critical Care Systems, Singapore) that relayed pressure data through an analog-to-digital converter onto a lab computer. Custom designed software recorded ICP in real time and stored data for later analysis[26,27]. Also attached to the transducer was a syringe column that contained artificial CSF (148.2 mM NaCl, 3.0 mM KCL, 1.4 mM CaCl_2_·6H_2_O, 0.8 mM MgCl_2_·6H_2_O, 1.5 mM Na_2_HPO_4_·7H_2_0 and 0.2 mM NaH_2_PO_4_·H_2_O; all chemicals from Sigma-Aldrich, St. Louis, MO). The level of artificial CSF was maintained at the animal’s head level during baseline pressure measurements (the middle point of an imaginary line connecting the base of two ears was considered as head level). ICP was manipulated by manual elevation and declination of the artificial CSF column. Various modifications of this basic setup were tested before adopting the final working model detailed in the results section. All animals used for the experiments were individually housed in specially designed cages. These cages were taller and had tops with enough space to allow passage of tubing to minimize chances of entanglement of the head gear. For connecting the cannula to PE tubing, animals were anesthetized using an isoflurane vaporizer (Airco Inc., Madison, WI). 

### Histology

At the end of each experiment, the rat was sacrificed by intraperitoneal sodium pentobarbital injection (Fatal-Plus, Vortech Pharmaceuticals Ltd., Dearborn, MI). Eyes were enucleated and placed in 10% neutral buffered formalin (Fisher Scientific Company, Kalamazoo, MI). The head was removed from the base of the foramen magnum and completely immersed in 10% neutral buffered formalin. After a week of fixation at 4°C, the brain was removed from the skull and placed in 10% neutral buffered formalin. Rat brains were dehydrated in a series of ascending ethanol concentrations (70, 80, 95 and 100%), cleared in xylene and embedded in paraffin. Tissue embedded paraffin blocks were sectioned at 5 μm (for eyes) or 6 μm (for brains) and mounted on glass slides (Superfrost/Plus; Fisher, Pittsburgh, PA). Sections were deparaffinized in xylene, rehydrated in descending series of ethanol concentrations (100, 95, 80 and 70%), rinsed in running distilled water, stained with hematoxylin (Electron Microscopy Sciences, Hatfield, PA), washed again in running tap water, counterstained with eosin (Richard Allan Scientific, Kalamazoo, MI) and dehydrated in a series of ascending ethanol concentrations (70, 80, 95 and 100%). Stained sections were immersed in xylene and mounted with Surgipath micromount medium (Surgipath Medical Industries, Richmond, IL). Images of tissue sections were taken with either an Olympus BH-2 light microscope (Center Valley, PA) or an Olympus dissecting microscope (model SZX16). Digital images were captured using a scope mounted DP73 digital camera (Olympus) and the Cellsens standard v1.6 software (Olympus). 

### Statistics

Pressure data collected at the end of every minute were averaged over a 60 minute period to obtain a one hour pressure reading. Average ICP was calculated as the mean ICP of all the 1 hr recordings for each artificial CSF column position. ICP at baseline and with column lowered or raised at various positions were compared using Student’s unpaired t-test.

## Results

### Selection of appropriate rat strain for use in the intraventricular cannula model

Both Sprague Dawley and Brown Norway rats were able to maintain the cannula in the lateral ventricles. Brown Norway rats (n=5) maintained the cannula for approximately 4 weeks while Sprague Dawley rats (n=3) were more active and less compliant to tethering. In 2 of the 3 Sprague-Dawley animals, the cap was dislodged within one week. When the cannula was inserted into the cisterna magna, Sprague Dawley animals (n=3) survived the surgery but similar to animals with lateral ventricle placement did not tolerate the cannula and removed the head gear. Surgical placement of the cannula into the cisterna magna of Brown Norway rats (n=6) resulted in decreased survival and increased neurological compromise. Based on the ability to maintain an implanted cannula for at least 4 weeks with minimal surgical issues, the Brown Norway rat with a stainless steel cannula inserted into the lateral ventricle became the animal of choice for all subsequent experiments.

### Monitoring ICP in lateral ventricle of Brown Norway rats

Initial studies were conducted on Brown Norway rats surgically implanted with a lateral ventricle cannula with an external 22 gauge 1.5 mm post. Use of silastic PE90 tubing was unsuccessful due to the inability to sustain attachment to the cannula. Based on this, a guide cannula with an external post that was longer (3 mm instead of 1.5 mm) with a larger bore size (20 gauge instead of 22 gauge) was tested. The longer external post and increased bore size enabled the use of plastic PE60 tubing which provided a tighter and more secure connection to the cannula while also reducing the elasticity effect on resistance and pressure due to increased compliance of the material. The PE60 tubing attached to the cannula was connected in parallel with an artificial CSF column and pressure transducer enabling real-time ICP recording.

### Modulation of ICP using the rat IVC model

**Figure 2:**
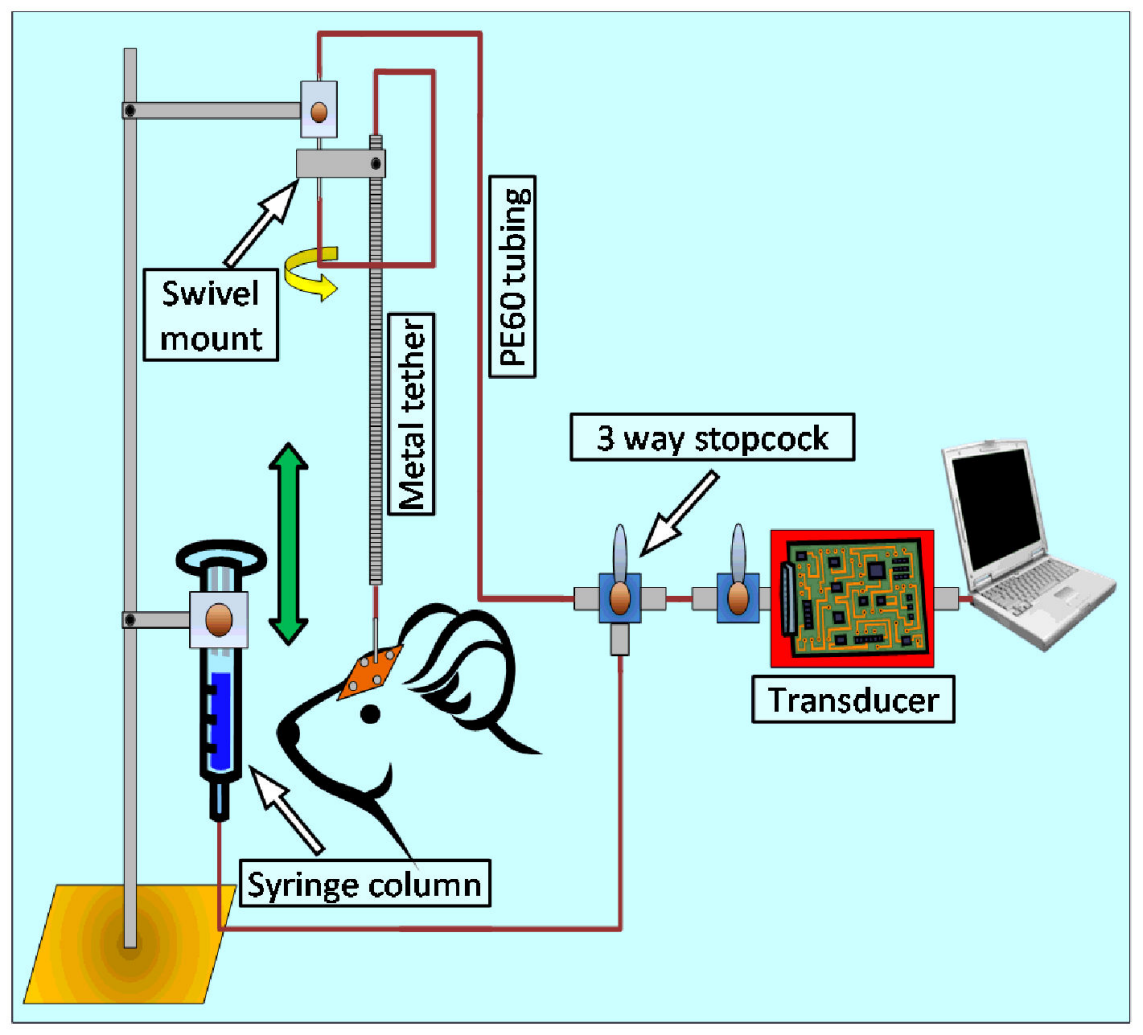
Detailed schematic diagram of the rat IVC model. The 3 mm external post of the guide cannula was connected to PE60 tubing protected by a metal tether. The tether connected to a swivel mount outside the cage to allow free movement of the animals. The PE60 tubing continued through a connector inside the swivel mount and attached to a pressure transducer via a 3-way stopcock. The data from the pressure transducer was collected and analyzed by custom designed software. A column containing artificial CSF was attached in parallel to the transducer through the same 3-way stopcock. Manual lowering or raising of the column decreased or increased ICP.

**Figure 3:**
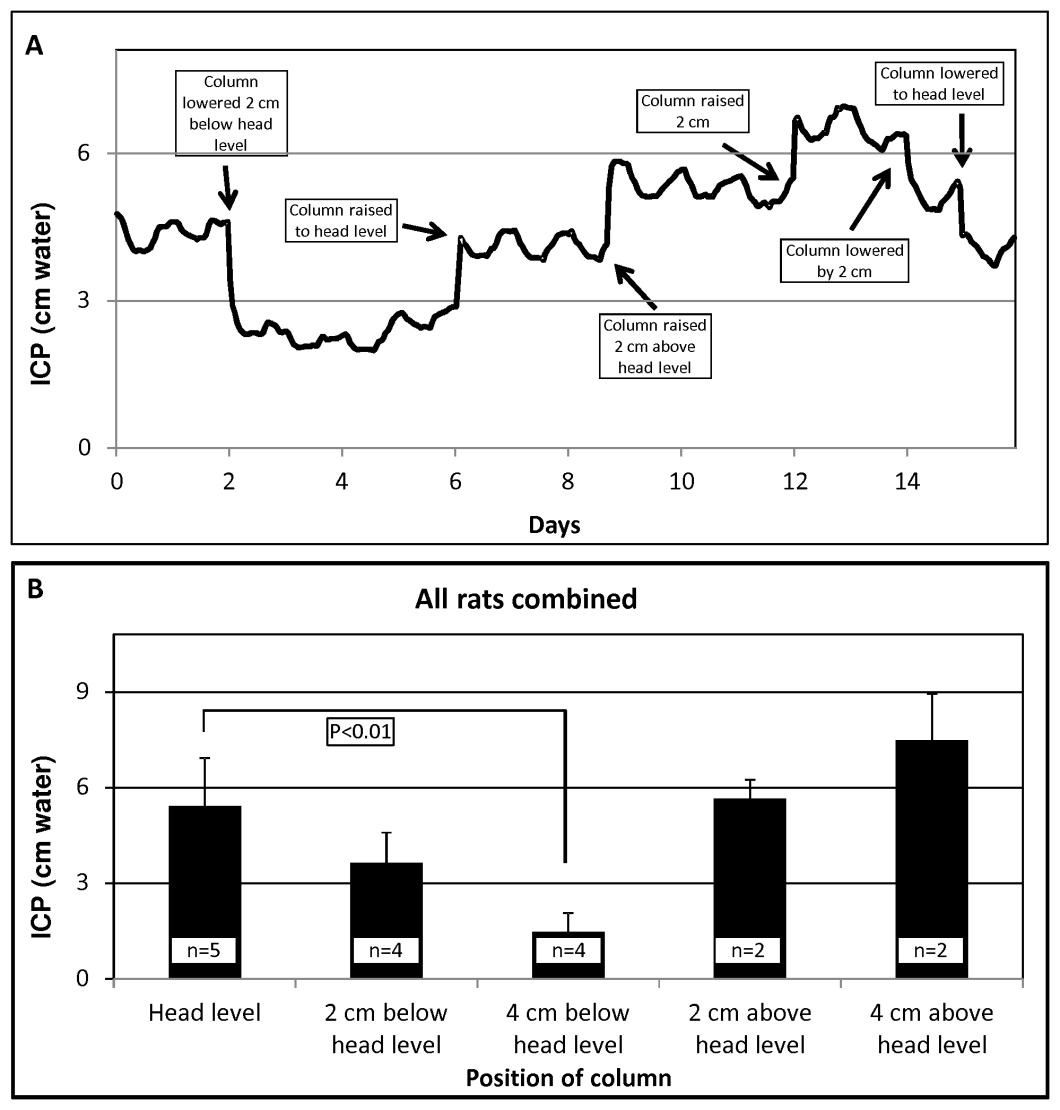
Changes in ICP due to manual manipulation of artificial CSF column. A) Representative ICP graph annotated with boxes and arrows to mark the points where the artificial CSF column was lowered or raised with reference to the animal’s head level. Lowering or raising of the artificial CSF column caused corresponding decrease or increase of ICP. B) Average ICP of all animals, with the artificial CSF column at various positions.

The final design of the IVC model consisted of a stainless steel cannula implanted in the lateral ventricle and connected externally to PE60 tubing attached to an artificial CSF syringe column positioned with the top level of the artificial CSF fluid at head level of the rat (Fig. 2). Use of a 3-way stop cock enabled the artificial syringe column to be attached in parallel with the pressure transducer. To evaluate ICP modulation, the artificial CSF column was lowered or raised by 2-cm increments (Figure 3, A). Results from 5 individual animals are shown in Table 1. Overall, average ICP at head level was 5.5±1.5 cm water (4.2 to 7.9 cm water, n=5). Positioning of the artificial CSF column by 2 or 4 cm below head level reduced ICP by 33.0% (range of reduction 25.0% to 44.8% with an overall average ICP reduction of 1.8 cm water) and 72.6% (range of reduction 62.9% to 84.8% and an average ICP reduction of 4.0 cm water) when compared to baseline ICP (Figure 3, B). Raising the column above head level by 2 cm showed a modest increase in ICP (4.6%, increase of 0.2 cm water). Repositioning of the artificial CSF column 4 cm above head level (n=2) resulted in a 38.3% increase in ICP (average ICP increase of 2.0 cm water; Figure 3, B). Attempts at further raising or lowering the column resulted in animal discomfort (n=2) as evident from lack of movement and noticeable change in head posture, presumably in an effort to compensate for significant changes in CSF pressure. 

**Table 1:** ICP readings from each rat at various artificial CSF column positions (values for .

	**ICP (cm water) at various column positions with respect to rat head (mean±standard deviation of all recorded timepoints for each rat with the column at any given position)**				
	**Baseline (Column at head level)**	**2 cm below head level (% change compared to baseline)**	**4 cm below head level (% change compared to baseline)**	**2 cm above head level (% change compared to baseline)**	**4 cm above head level (% change compared to baseline)**
**Rat 1**	4.5±0.5	N/A	0.7±0.4 (-84.8%)	N/A	N/A
**Rat 2**	7.9±0.7	4.4±0.4 (-44.8%)	1.5±0.5 (-81.0%)	N/A	N/A
**Rat 3**	4.8±0.1	3.4±0.0 (-28.6%)	1.8±0.1 (-62.9%)	6.1±0.0 (28.6%)	8.6±0.0 (80.0%)
**Rat 4**	6.0±0.1	4.5±0.5 (-25.0%)	2.0±0.4 (-65.9%)	N/A	N/A
**Rat 5**	4.2±0.3	2.4±0.3 (-41.9%)	N/A	5.3±0.3 (25.8%)	6.5±0.3 (54.8%)
**Average±SD**	5.5±1.5	3.7±0.9 (-35.1±9.8%)	1.5±0.6 (-73.7±10.9)	5.7±0.6 (27.2±2.0)	7.5±1.4 (67.4±17.8%)

### Histologic analysis of the brain

**Figure 4:**
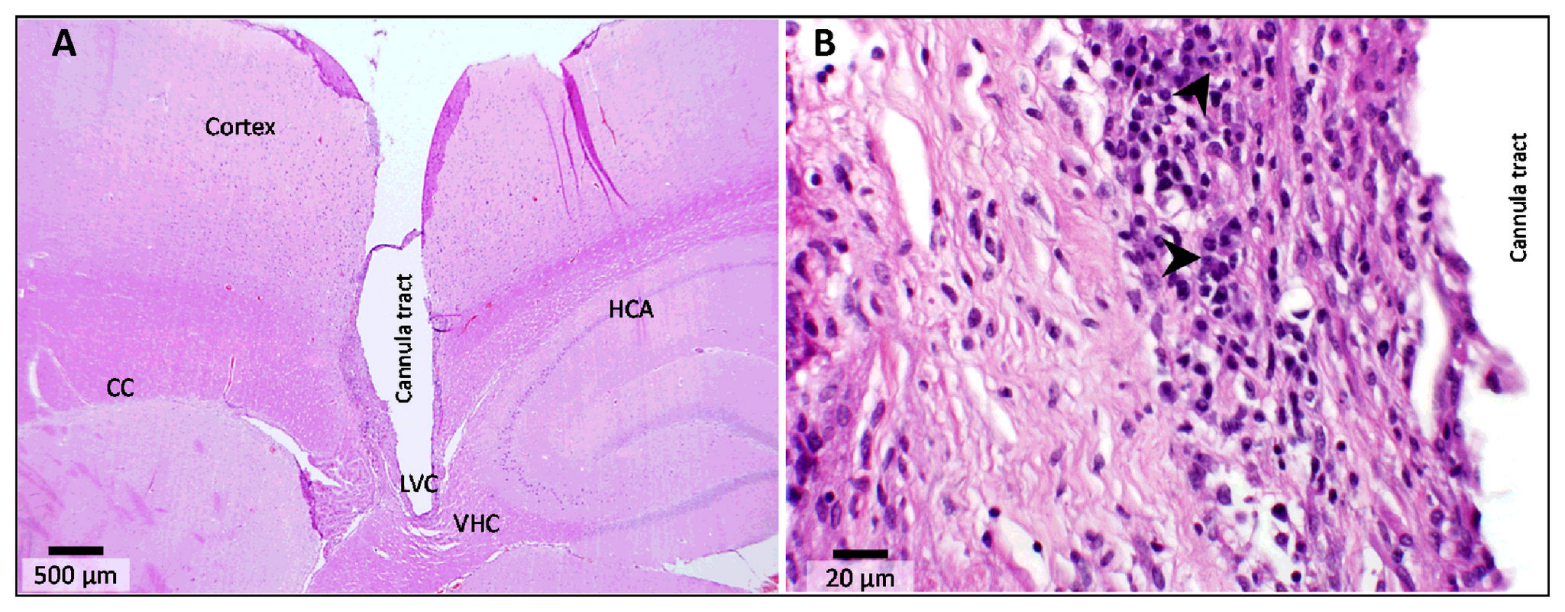
Histology of cannulated lateral ventricle. A) Cross-section through the rat brain shows a clean tract of the cannula and relatively healthy tissue with little or no inflammation. B) Representative micrograph from rat 3 showing signs of a delayed immune reaction, as evident from the clusters of plasma cells (arrow heads). This was presumed to be a response to the implanted cannula. CC, corpus callosum; HCA, hippocampus (Ammon’s horn); LVC, left ventricle; VHC, ventral hippocampal commissure.

Histologic sections of the rat brains (n=5) showed a clean cannula tract through the cerebral cortex extending into the left lateral ventricle and minimal invasive damage to brain tissue (Figure 4, A). Overall, there appeared to be minimal inflammation due to cannula placement. Only rat 3 showed migration of plasma cell clusters indicating a delayed immune reaction probably in response to the cannula (Figure 4, B). 

## Discussion

Almost 90 years ago, investigators first speculated that ICP may have a role in modulating optic nerve changes associated with glaucoma[28]. More recent studies have confirmed an association between reduced ICP and POAG and NTG, suggesting ICP as a risk factor for glaucoma[4,5,9,10,15–18,21,22,29–31]. Unfortunately, the ability to assess the association between ICP and glaucoma has been difficult to study in a controlled environment because of the absence of a suitable animal model. The rat IVC model is the first comprehensive animal model where ICP can be manually reduced or increased over an extended period of time to study the balance between IOP and ICP and its role in optic nerve pathology. 

ICP is the result of a balance between CSF formation and drainage[32]. In human patients, chronic elevation of ICP without any underlying cause is believed to be a result of CSF outflow abnormality[33]. On the other hand spontaneous intracranial hypotension may be caused by a leak of CSF[33]. In the IVC model, we believe that the ICP is lowered by outward drainage of CSF possibly due to a siphon effect[34], when the artificial CSF column is lowered. On the contrary, ICP would be raised by infusion of artificial CSF from the attached column based on a pressure difference, when the column is raised. Nevertheless, based on available data on CSF outflow, rats appear to be a good choice for a model animal, due to similarities in CSF drainage pathways with humans[35–37].

. Several reports indicate that pressure in the subarachnoid space is equivalent to the CSF pressure. When measured by lumber puncture, CSF pressure has been found to correlate well with the ICP as well as the retrolaminar pressure, measured in the lateral decubitus position. Because of this, ICP and CSF pressure have been used interchangeably in published literature as well as in clinical practice[4,5,11,12,18,31,38,39]. The IVC model had an effective ICP modulation range of 4 cm above or below head level. This is equivalent to an ICP change of +38.3% to -72.7%, within the range of ICP change for POAG (-23%) and NTG (-26%)[17]. Rats appeared to sense a change in ICP arising from alterations of the column height. Whenever the column height was adjusted, the rats tried to keep their head raised or lowered to compensate for the change in pressure. This was particularly evident when the column was raised by 2 cm resulting in a modest change in ICP. More significant and consistent results were obtained with movement of the column by 4 cm. However, once the column was moved greater than 4 cm from head level, the rats showed discomfort indicating a 4 cm change was the maximal distance to move the column while maintaining the least amount of animal discomfort.

The cannula placement in the lateral ventricle and subsequent manipulation of ICP were well tolerated by Brown Norway rats. Increased mortality and neurological complexities were obtained in Brown Norway rats with cannula placement in the cisterna magna. Although the reason behind this was unclear, we suspect this to be a result of a stronger host body reaction to implanted foreign material in the cisterna magna as has been reported in dogs[40]. This prompted us to select the lateral ventricle as the preferred site of cannula implantation. We did not encounter any morbidity or mortality from ICP manipulations within the tolerable range as discussed above. A small number of animals have succumbed within a few days of initiating ICP recordings. However, we believe this was due to stress from the cannula implantation surgery rather than the experiment itself. Several other animals failed to record a stable baseline and were also not included in the study. The main reason for this was also surgical in nature due to tissue clogging the cannula. Overall, in maintaining a stable baseline and at least one cycle of artificial CSF column manipulation, we achieved a 60% success rate. 

Previous animal models have utilized CSF shunts to lower ICP[41–43]. Two abstracts, written over 30 years ago, assessed the role of ICP reduction in cats using cannulation of the cisterna magna (Yablonski ME, et al. *IOVS* 1978;17:ARVO Abstract 6; Yablonski ME, et al. *IOVS* 1979;18:ARVO Abstract 8). The authors never formally published their findings and therefore it is difficult to evaluate the merits of their model. However, they did report optic nerve changes, including prelaminar axonal swelling and cupping of the lamina cribrosa, when ICP was lowered below atmospheric pressure for up to 7 days. 

Intracranial hypertension has been created using pharmacologic agents (e.g. kaolin or GABA receptor antagonists)[44–46] or by infusion of artificial CSF into the lateral ventricle[47,48]. Unfortunately, in these studies pressure could only be measured for a few minutes to a few hours with the animal often under anesthesia[45,48]. Additionally, the pressure changes were short term and may not be suitable for modeling glaucoma. In the IVC model, lowering of ICP was achieved in non-anesthetized animals for as long as 15 consecutive days. Studies analyzing extended duration of ICP modulation are currently being performed.

The IVC model allows maintenance of a lowered ICP for several days, as opposed to acute changes for a few hours. This has tremendous implications in studying ocular pathologies like glaucoma where a chronic change in ICP (with or without an elevated IOP) may have a detrimental effect on optic nerve health. The model also enables investigation into the relationship between ICP and IOP and the translaminar pressure differential. Modulation of both ICP and IOP appears to occur in the dorsomedial/perifornical region of the hypothalamus [45] suggesting that both IOP and ICP are interlinked with reference to their physiologic significance. Additionally, this model can also be used to study systemic disorders arising from intracranial hypo- and hypertension (e.g. intracranial hypotension syndrome, stroke, cardiac arrest). While intracranial hypotension generally presents with mild to severe headaches, elevated ICP can often cause fatal trauma to the brain through various acute episodes of stroke and cardiac arrest. The IVC model may be used to evaluate the basic mechanisms behind these pathologic conditions. Current pharmaceuticals used to alter ICP may also be examined for mode of action. 

In conclusion, the rat IVC model can be successfully used to measure ICP over sustained periods of time while the attached artificial CSF column enables the investigator to lower or raise ICP in a controlled fashion. Future studies directed at understanding the interplay between IOP and ICP will open new avenues in our understanding of optic neuropathies and the rat IVC model can be an invaluable tool in bridging the current knowledge gap. The rat IVC model offers a viable method to study the effect of intracranial hypertension and hypotension on various ocular (glaucoma, papilledema) as well as systemic pathologies (stroke, brain injury, cardiac arrest, etc.) where ICP plays an important role.
